# Editorial: Programmed cell death in aquatic animals

**DOI:** 10.3389/fimmu.2024.1428742

**Published:** 2024-06-14

**Authors:** Qingchao Wang, Dahai Yang, Linlin Zhang, Madison Powell, Yu-Hung Lin

**Affiliations:** ^1^ Department of Aquatic Animal Medicine, College of Fisheries, Huazhong Agricultural University, Wuhan, China; ^2^ State Key Laboratory of BIoreactor Engineering, East China University of Science and Technology, Shanghai, China; ^3^ Key Laboratory of Experimental Marine Biology, Institute of Oceanology, Chinese Academy of Sciences (CAS), Qingdao, China; ^4^ Aquaculture Research Institute, University of Idaho, Moscow, ID, United States; ^5^ Department of Aquaculture, National Pingtung University of Science and Technology, Pingtung, Taiwan

**Keywords:** programmed cell death, apoptosis, necroptosis, FAS/FASL, MLKL

Programmed cell death (PCD) is an evolutionarily conserved cell suicide that functions in tissue growth regulation, cell turnover, immune response, and other biological processes. Aquatic animals are continuously exposed to fluctuating physicochemical factors, enriched pathogenic bacteria, and unbalanced nutrient supply in the water environment, and programmed cell death is coordinated with the immune system to maintain tissue homeostasis in aquatic animals. This Research Topic collects 5 original research articles to identify the key proteins involved in programmed cell death.

Apoptosis is the most well-studied type of PCD, which plays a key role in the immune system, and it can function via an exogenous pathway or an endogenous pathway. In particular, pathogenic infections can activate the extrinsic pathway via the death receptor superfamily, which includes the Fas/FasL system. In this specific Research Topic, Qin et al. (2023) identified a novel FasL gene from *Crassostrea hongkongensis* that exhibited typical characteristics of the TNF family. ChFasL, which is located in the cytoplasm, is involved in the immune response to external microbial stimulation and also exerts a pro-apoptotic effect. Apoptosis is also regulated by immunostimulants such as β-Glucans, although the regulatory role varies depending on structural variations and dosage. Wu et al. (2023) indicated that treatment with insoluble Paramylon at high doses resulted in significant apoptosis, whereas soluble Laminarin did not induce apoptosis even at high doses. Moreover, the intrinsic apoptotic pathway was responsible for the apoptosis induced by high-dose Paramylon, while Laminarin triggered metabolic reprogramming by promoting a-Ketoglutarate production to protect the macrophages from apoptosis.

In addition to apoptosis, necroptosis is a new type of proinflammatory programmed necrosis that is essential for innate immunity. Hao et al. (2023) identified the receptor-interacting protein kinases 1/3 (RIPK1/3) and mixed lineage kinase domain-like protein (MLKL) from *Paralichthys olivaceus* in the necroptotic axis. PoRIPK1/3 interacted with PoMLKL via the RIP homotypic interaction motif (RHIM) to enhance the necroptosis-inducing activity of the N-terminal four-helix bundle (4HB) domain in PoMLKL. Moreover, PoMLKL-mediated necroptosis contributed to the defense against *Edwardsiella tarda* infection in fish cells and tissues. In another paper, Yu et al. (2024) indicated that IFN-γ enhanced the protective efficacy against *Nocardia seriolae* infection in largemouth bass (*Micropterus salmoides*), and also demonstrated the transformation of granuloma status from an early necrotic foci to fibrosis during the infection period by histopathological examination.

In summary, this Research Topic delivers new information for ongoing research on programmed cell death in aquatic animals. Programmed cell death exhibited a co-coordinating role with immune responses in aquatic animals under different circumstances. Shi et al. (2024) also revealed the novel mechanism of DNA methylation in B cell activation via repression of Pax5 expression in teleosts. We thank all the authors for their contributions and hope that our Research Topic will stimulate and deepen the knowledge of programmed cell death in aquatic animals.

## Author contributions

QW: Writing – original draft, Conceptualization. DY: Writing – review & editing, Conceptualization. LZ: Writing – review & editing, Conceptualization. MP: Writing – review & editing, Validation. Y-HL: Writing – review & editing, Validation.

